# Ginkgo Biloba for Alzheimer’s Disease: From Mixed Dementia Trials to Biomarker-Confirmed Mild Cognitive Impairment—What Have We Learned over Two Decades, and Is There Finally a Bit of Hope?

**DOI:** 10.3390/brainsci16040430

**Published:** 2026-04-20

**Authors:** YoungSoon Yang, Yong Tae Kwak

**Affiliations:** 1 Department of Neurology, Soonchunhyang University Cheonan Hospital, 31 Soonchunhyang 6-gil, Dongnam-gu, Cheonan-si 31151, Chungcheongnam-do, Republic of Korea; astro76@naver.com; 2 Department of Neurology, Hyoja Geriatric Hospital, 1-30, Jungbu-daero 874beon-gil, Giheung-gu, Yongin-si 17089, Gyeonggi-do, Republic of Korea

**Keywords:** Ginkgo biloba, Alzheimer’s disease, amyloid PET, mild cognitive impairment, MDS-OAβ

## Abstract

Ginkgo biloba products have been used for decades for cognitive symptoms, yet the clinical evidence in Alzheimer’s disease (AD) remains modest and heterogeneous. This review revisits key symptomatic and prevention trials and summarizes how systematic reviews and meta-analyses have informed ongoing clinical skepticism, often citing small effect sizes, limited patient-centered meaningfulness, short follow-up, and repeated trial designs. We suggest that long-standing ambiguity reflects multiple, overlapping sources of heterogeneity, including mixed-pathology recruitment, variable dosing and exposure duration, inconsistent outcome frameworks, and limited integration of biological readouts; differences across preparations and characterization practices may further contribute to variability. In the biomarker era, AD is increasingly defined biologically, and amyloid PET-confirmed cohorts offer a clearer test by reducing diagnostic noise and enabling mechanism-adjacent interpretation. Recent studies in amyloid PET-positive MCI/AD report clinical preservation alongside directional changes in plasma oligomerization tendency (MDS-OAβ), with decreases in treated groups compared with increases in controls. While such findings cannot, by design, establish disease-modifying effects, they provide a biologically anchored context for interpreting modest clinical signals. We conclude with practical recommendations to align cohort biology, stage, exposure certainty, duration, endpoints, and biomarker panels in next-generation trials of Ginkgo preparations in early AD-spectrum disease.

## 1. Introduction

Alzheimer’s disease (AD) remains the most common cause of dementia and continues to impose a major burden on patients, caregivers, and healthcare systems [1]. Even as anti-amyloid immunotherapies reshape parts of the treatment landscape [2], practical clinical questions remain unresolved: what can be offered to patients who do not qualify for—or cannot access—disease-targeting therapies; what adjunctive approaches might plausibly stabilize cognition, function, or neuropsychiatric symptoms; and how should clinicians interpret decades of botanical research that is extensive, popular, and yet persistently ambiguous?

In this review, we first take that “extensive but ambiguous” literature seriously: we examine the major symptomatic-treatment trials and prevention studies, then synthesize how systematic reviews and meta-analyses have shaped clinical skepticism, and finally re-evaluate the field through the lens of contemporary AD biology. Ginkgo biloba has been studied for decades across dementia syndromes and AD, including landmark placebo-controlled trials and subsequent AD-focused randomized studies [3,4]. Yet it also attracts a particular frustration. A recent review of randomized trials in mild-to-moderate dementia asked what has been learned over the past 18 years and concluded “not much” [5]. The authors emphasized that changes in measures such as the MMSE, SKT, and NPI were often small and frequently below thresholds many clinicians would consider clearly clinically meaningful, and they also noted recurring design constraints such as short follow-up and the dominance of placebo-controlled comparisons. This skepticism has been reinforced by prevention-era experience: large community-based trials in older adults did not reduce incident dementia/AD or meaningfully slow cognitive decline compared with placebo [6,7].

However, it is also important to recognize that literature has not been uniformly negative—especially in more recent syntheses that focus on clinically relevant domains beyond cognition alone. For example, trials enriched for neuropsychiatric symptom burden, or those using higher daily dosing over months, have reported improvements across cognition, behavioral symptoms, and daily functioning [8,9]. More recent meta-analyses have continued to report favorable pooled effects in mild dementia on cognition and activities of daily living, while emphasizing heterogeneity and study quality [10,11,12]. In addition, an overview of systematic reviews synthesized the broader EGb 761 evidence base across indications and highlighted that conclusions require caution given the methodological limitations of many reviews [13]. Real-world observational analyses have also reported associations between prescriptions of Ginkgo leaf extract and reduced risk of dementia severity progression, albeit with the inherent limitations of non-randomized designs [14]. Taken together, these “favorable” signals are real in the published record, but they coexist with substantial uncertainty about who benefits, how durable the effects are, and whether observed changes translate into meaningful patient-centered outcomes.

**Figure 1:**
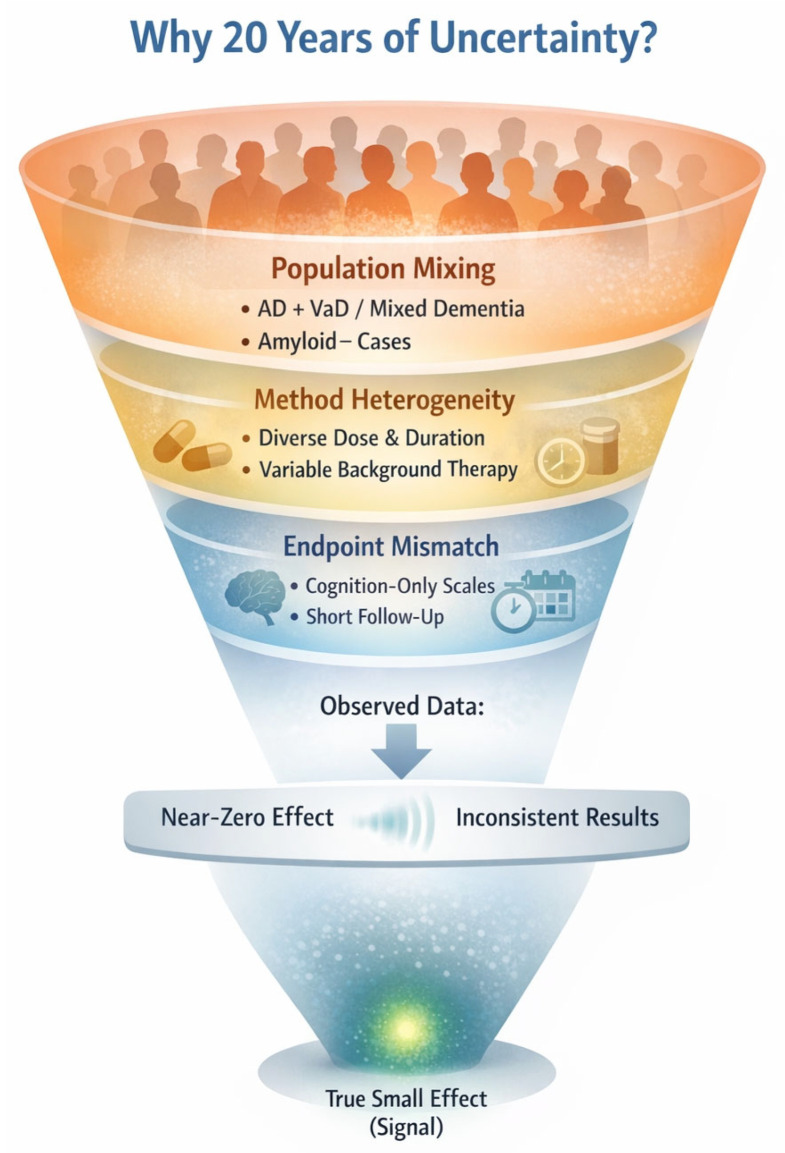
Conceptual model of signal dilution in the historical Ginkgo–Alzheimer’s disease literature. This schematic summarizes “signal dilution” in the historical Ginkgo–AD literature. Population mixing (e.g., mixed/vascular dementia and amyloid-negative cases), methodological heterogeneity (dose, duration, background therapy, design), and endpoint mismatch (cognition-only scales, short follow-up, limited functional/caregiver outcomes) can obscure a small true effect, yielding near-zero or inconsistent results. The model motivates biomarker-enriched, stage-appropriate trial designs to improve interpretability.

The core interpretive problem is not which named preparation was used, provided that the intervention was pharmaceutical-grade and adequately characterized [15]. The main barrier has instead been layered heterogeneity in both disease biology and trial methodology (Figure 1). First, the meaning of “AD” has evolved. Many older trials enrolled patients based on syndromal criteria (probable AD; dementia of Alzheimer type) and often mixed AD with vascular or “mixed dementia,” while contemporary frameworks increasingly define AD as a biological entity using biomarkers of amyloid, tau, and neurodegeneration [16]. If modest benefits are concentrated primarily within biologically confirmed AD-spectrum disease, then syndromal recruitment and mixed-pathology cohorts would be expected to dilute treatment signals. Second, methodological heterogeneity—dose and exposure duration, baseline severity, neuropsychiatric symptom burden, background therapy, comparator strategy, and endpoint selection— may also have contributed importantly to “signal dilution,” helping to explain why meta-analyses can suggest benefits for cognition and function while clinicians remain unconvinced about clinical meaningfulness [5,10,11]. Third, outcome frameworks have often prioritized short-term cognitive deltas rather than integrated cognitive–functional outcomes and caregiver-relevant endpoints, making it difficult to judge whether statistically significant changes are truly meaningful in daily life [5]. A further source of ambiguity is that much of the historical program was scale-driven rather than biology-driven [17], limiting insight into why modest clinical changes may have occurred. In practice, earlier attempts to incorporate biomarkers were either indirect (e.g., peripheral inflammatory/oxidative stress panels in MCI) or observational analyses of blood amyloid measures in large prevention cohorts, rather than trials explicitly enriched for AD biology [18,19]. Consequently, until very recently, the field had few opportunities to interpret Ginkgo’s clinical signals through a mechanism-adjacent, AD-confirmed lens—precisely the gap that biomarker-confirmed designs (amyloid PET-positive MCI/AD) are now positioned to address.

**Figure 2:**
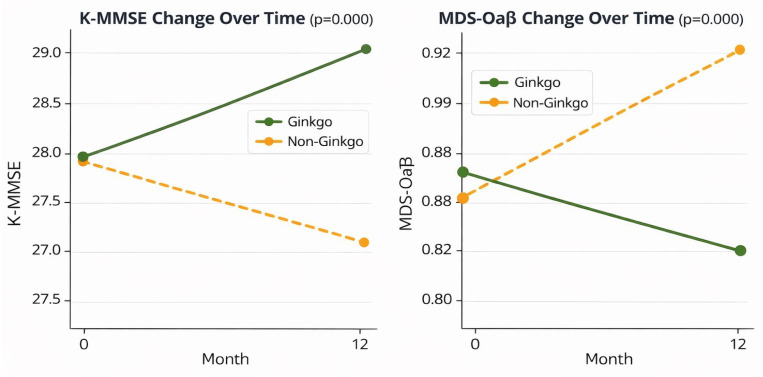
Twelve-month changes in global cognition (K-MMSE) and plasma MDS-OAβ in amyloid PET-positive MCI. Line plots illustrate changes from baseline to 12 months in Korean Mini-Mental State Examination (K-MMSE) scores and plasma Multimer Detection System–Oligomerized Aβ (MDS-OAβ) levels in amyloid PET-positive mild cognitive impairment (MCI) patients treated with Ginkgo biloba (240 mg/day) versus non-Ginkgo cognitive enhancers. The Ginkgo group demonstrated slight cognitive improvement and a reduction in plasma MDS-OAβ, whereas the non-Ginkgo group showed cognitive decline and an increase in MDS-OAβ over the same period. Between-group differences in change were statistically significant for both measures (*p* < 0.001). This figure was adapted from [20].

Against this background, biomarker-confirmed studies in amyloid PET-positive MCI/AD constitute a qualitatively different test of the hypothesis because they reduce diagnostic noise and incorporate mechanism-adjacent biomarker outcomes. In amyloid PET-positive MCI, Ginkgo monotherapy has been reported to preserve cognition, improve daily function, and reduce plasma measures related to amyloid oligomerization (Figure 2) [20,21]. In amyloid PET-positive AD, adjunct-to-donepezil designs have reported aligned clinical outcomes alongside changes in a plasma oligomerization-related biomarker [22]. These studies do not establish disease modification and require larger, prospective replication. At present, they should be regarded as preliminary and hypothesis-generating, suggesting that some of the field’s long-standing ambiguity could reflect the way earlier studies defined populations and outcomes. Accordingly, this review revisits the historical evidence base with explicit attention to heterogeneity and methodological design, and then examines biomarker-confirmed AD-spectrum studies as a potential pivot point. While the overall message over ~20 years may remain modest, there may be a bit of hope if future research avoids mixing biologically disparate populations and aligns dose, duration, endpoints, and biomarkers with disease stage (Table 1).

## 2. Scope and Approach

This review begins with a straightforward premise: the Ginkgo biloba literature in cognitive disorders is extensive, but its clinical interpretation in Alzheimer’s disease (AD) remains unsettled. We therefore first revisit the major bodies of evidence that shaped current skepticism-classic symptomatic trials, prevention trials, and the systematic reviews/meta-analyses that summarized small-to-modest effects with substantial heterogeneity—before turning to the recent shift in AD research toward biomarker-defined disease and stage. The aim is not to re-run meta-analytic calculations, but to synthesize why results diverged historically and which design features are most likely to determine whether a reproducible signal can be detected in AD-spectrum populations [5,23,24].

Our disease focus is AD, while acknowledging that many influential trials enrolled mixed dementia cohorts (AD with vascular or “mixed” dementia) and must be interpreted accordingly. Evidence considered includes randomized controlled trials (symptomatic and prevention), head-to-head and add-on/combination studies with established symptomatic therapies, and systematic reviews/meta-analyses that have repeatedly highlighted heterogeneity in populations, dosing strategies, follow-up duration, and endpoint selection [10,11,23]. Within these categories, studies were prioritized for discussion based on their relevance to AD-focused clinical interpretation, their influence on the historical or current evidence landscape, and, where applicable, their use of biomarker-defined cohorts or biomarker-linked outcome measures. Studies not directly relevant to AD-spectrum clinical interpretation, lacking sufficient methodological detail, or focused primarily on poorly characterized non-standardized exposure are not discussed in detail. In addition, we consider recent biomarker-confirmed studies that may offer a more biologically interpretable framework than earlier clinically defined studies [16,20,21,22]. Importantly, we do not treat “one named extract versus another” as the dominant explanation for decades of ambiguity. Conversely, when preparation reporting is poor—particularly in the supplement literature—exposure uncertainty becomes an additional confounder that can further widen variability across studies [25]. In this context, we place special emphasis on biomarker-linked interpretation—using clinical outcomes alongside mechanism-adjacent measures—to move beyond “whether it works” toward “how and in whom it may work,” an approach that has been uncommon in earlier clinically defined, non-biomarker-confirmed AD-spectrum Ginkgo trial literature. Accordingly, this review does not aim to argue that Ginkgo biloba has established efficacy in AD; rather, it examines whether decades of inconsistency can be reinterpreted in light of biological case definition and biomarker-linked study designs.

## 3. What Is “Ginkgo Biloba” in Clinical Research?

In clinical research, “Ginkgo biloba” is best understood not as a single uniform intervention, but as a botanical source whose preparations vary in constituent profiles depending on plant material, extraction methods, and manufacturing controls. For evidence synthesis, the practical implication is not that one named product is uniquely valid and all others are not; rather, it is that studies must be interpreted through the lens of exposure certainty. Several systematic syntheses have emphasized that meaningful pooling depends on knowing what was actually administered, and that non-equivalence across preparations can add noise—especially when preparation characterization is incomplete [23,24]. At the same time, for an AD-focused clinician, preparation identity is rarely the dominant barrier when the intervention is pharmaceutical-grade and well described; historically, larger interpretive problems have usually come from heterogeneity in disease biology and heterogeneity in methodology (dose, duration, comparators, endpoint choice, and analytic framing of clinical meaning) [5,10,11].

Across the pivotal dementia/AD trial literature, standardized extracts were commonly administered within ranges such as 120–240 mg/day, often over ~22–26 weeks, although trial durations varied, and longer follow-up has been less frequent [23,24,26]. Such design features matter because short durations can test symptomatic benefit but are less suited to demonstrating durable changes in decline trajectory in progressive neurodegeneration. Mechanistic plausibility has persisted because Ginkgo preparations contain multiple compound classes, particularly flavonoid glycosides and terpene lactones, that are plausibly relevant to oxidative stress modulation, mitochondrial support, microcirculatory regulation, neuroinflammation-related pathways, synaptic resilience, and amyloid-related biological processes (Table 2) [27]. However, plausibility alone has not resolved clinical controversy: observed clinical effects have generally been modest and heterogeneous. Moreover, prevention trials in broad older populations were convincingly null for dementia incidence [5,6,7,24].

## 4. Symptomatic Trials in Alzheimer’s Dementia: What Did Classic RCTs Show?

The symptomatic trial era includes multiple randomized placebo-controlled studies, some enrolling AD-only cohorts and others combining AD with vascular or mixed dementia. A recurring pattern emerges: some trials report improvements over placebo in cognition, global impression, neuropsychiatric symptoms, and activities of daily living, but effect sizes vary, durability is uncertain, and clinical meaningfulness remains debated—especially when cognition-only deltas are small or when endpoints are not optimally aligned to stage and caregiver-relevant change [3,4,5,23].

A landmark early placebo-controlled trial evaluated a standardized Ginkgo extract in AD and multi-infarct dementia, helping establish feasibility and a plausible symptomatic signal while also illustrating an enduring limitation: mixed-etiology recruitment complicates AD-specific inference [3]. Later AD-specific randomized studies reduced etiologic noise relative to mixed cohorts. For example, a multicenter double-blind placebo-controlled trial tested two doses of Ginkgo extract in dementia of the Alzheimer type, representing an AD-focused effort to clarify efficacy under more homogeneous diagnostic framing [4]. Yet, even trials conducted in clinically diagnosed AD cohorts did not consistently yield large or unambiguous effects, contributing to cautious conclusions in later review [5,28]. This pattern underscores how modest true effects—if present—can remain equivocal when trial duration, endpoint selection, and population heterogeneity are not optimized

A particularly relevant sub-literature enriched patients for neuropsychiatric symptoms (NPS), a domain that frequently drives caregiver burden and institutionalization. In the GINDEM-NP study, a randomized double-blind placebo-controlled trial in mild-to-moderate dementia with neuropsychiatric features reported efficacy, supporting the notion that benefits—when present—may be more evident in behavioral symptoms and related global outcomes than in pure cognitive deltas [8]. Subsequent placebo-controlled work tested once-daily 240 mg dosing in dementia with neuropsychiatric symptoms and reported superiority versus placebo, again suggesting that phenotypic enrichment and endpoint choice can materially influence detectability [29]. A related placebo-controlled trial similarly emphasized improvements in cognition and neuropsychiatric outcomes in a target population defined by clinically relevant NPS, reinforcing the idea that “where the signal shows up” may depend on what is measured and who is enrolled [9]. Notably, the apparent prominence of benefit in these neuropsychiatric-symptom-enriched trials coincides with a higher-dose strategy. In both once-daily RCTs targeting dementia with neuropsychiatric features, 240 mg/day regimens were associated with statistically significant—and in at least one report, clinically relevant—improvements across cognition, psychopathology-related outcomes and functional measures [9,29]. While cross-trial comparisons cannot cleanly separate dose from population and endpoint differences, convergent meta-analytic findings also point to 240 mg/day as the regimen most consistently linked to efficacy signals in dementia [10,11].

Head-to-head and combination strategies have also been influential because they map onto real-world practice, where botanicals are often used adjunctively rather than as replacements. A randomized placebo-controlled double-blind study compared a Ginkgo extract with donepezil (and placebo) in AD and reported comparable clinical efficacy, though such comparisons are inherently sensitive to dosing equivalence, power, and blinding considerations [30]. An exploratory double-blind trial compared extract monotherapy, donepezil monotherapy, and combined treatment in AD with neuropsychiatric features, reflecting persistent clinical interest in add-on strategies and suggesting that combined regimens may yield differential outcomes in selected phenotypes [31]. Taken together, the symptomatic trial literature supports neither blanket dismissal nor confident endorsement; rather, it supports a cautious interpretation that small-to-modest benefits may exist in certain domains or subgroups, while substantial uncertainty remains regarding clinical meaningfulness and reproducibility across heterogeneous cohorts [5,10,23].

Taken together, the historical symptomatic RCTs are important, not because they establish robust efficacy, but because they define the limits of what could be inferred from clinically diagnosed and often heterogeneous dementia populations. Their syndromal or mixed-pathology recruitment strategies, modest trial durations, and variable endpoint frameworks likely made it difficult to determine whether small treatment signals were truly absent or instead diluted below detectability in the populations studied.

## 5. Prevention and Cognitive Decline Trials: What Did We Learn from Large Null Results?

The prevention literature has often functioned as the field’s strongest counterargument. In the large Ginkgo Evaluation of Memory trial, Ginkgo biloba did not reduce overall dementia incidence or AD incidence in older adults, a finding widely interpreted as evidence against population-level prevention [6]. Related work examining cognitive decline endpoints in broad older populations similarly reported no meaningful prevention of decline compared with placebo [7]. These results are important and should not be minimized: they argue strongly against recommending Ginkgo as a general preventive strategy for dementia/AD in unselected older adults. These large null prevention trials should be interpreted within their intended context: they were designed to test population-level prevention in broad older adult cohorts, not treatment responsiveness within biologically confirmed AD-spectrum disease. They are therefore highly informative negative studies, but they do not fully resolve whether a modest signal might still be detectable in biologically defined, early-stage AD populations. At the same time, prevention nulls do not automatically resolve the narrower AD-clinic question addressed in symptomatic or early-stage trials. A modest symptomatic effect, or a stage-limited benefit in biologically defined AD-spectrum disease, might not translate into reduced incident dementia in broad populations over long follow-up. Moreover, if benefits—if any—are contingent on amyloid positivity or other biomarker-defined substrate, trials enrolling broad older cohorts would be expected to dilute detection. This is not wishful thinking so much as a testable hypothesis that becomes empirically addressable in biomarker-enriched designs [6,7,16].

## 6. Systematic Reviews and Meta-Analyses: Why Conclusions Diverged

Multiple systematic reviews/meta-analyses have attempted to reconcile heterogeneity, and their conclusions often converge on a familiar theme: possible benefit versus placebo in cognition and/or function, but uncertainty driven by heterogeneity and variable trial quality. A widely cited systematic review/meta-analysis in BMC Geriatrics reported a pooled cognitive effect favoring Ginkgo over placebo (SMD −0.58, 95% CI −1.14 to −0.01, *p* = 0.04), whereas the pooled effect on activities of daily living did not reach statistical significance (SMD −0.32, 95% CI −0.66 to 0.03, *p* = 0.08), while heterogeneity across trials remained substantial [23]. Later meta-analytic work likewise reported favorable pooled effects, but with important differences in outcome domains, extract definition, and interpretive emphasis [10,11,24]. In particular, Tan et al. reported significant pooled benefits of EGb 761 on cognition (WMD −2.86, 95% CI −3.18 to −2.54, *p* < 0.00001), activities of daily living (SMD −0.36, 95% CI −0.44 to −0.28, *p* < 0.00001), and global clinical change (Peto OR 1.88, 95% CI 1.54 to 2.29, *p* < 0.00001), with the strongest effects observed at 240 mg/day [10]. An extract-defined systematic review/meta-analysis similarly concluded efficacy and good tolerability for a defined standardized extract, while emphasizing that meaningful synthesis requires clear preparation definition because interchangeability across marketed products cannot be assumed [24]. A Cochrane review likewise concluded that Ginkgo may be better than placebo on global outcomes, cognition, and activities of daily living at around six months, but emphasized substantial between-study heterogeneity and therefore limited certainty [32,33]. Taken together, these syntheses suggest that any symptomatic benefit—when detected—tends to be most apparent under specific study conditions, including more clearly defined populations, higher standardized dosing (often 240 mg/day), sufficient exposure duration (≈6 months), and outcome frameworks that extend beyond cognition alone to include function and neuropsychiatric symptoms (Table 3). However, even with these patterns, the overall evidence remains difficult to translate into consistently “clinically meaningful” change at the individual patient level. Accordingly, the recent “not much” critique represents a shift in emphasis: rather than debating statistical significance, it foregrounds clinical meaningfulness thresholds and argues that many observed changes are small and that repeated similar trials may not be justified without a design shift [5]. This framing is valuable, but it is also historically anchored: it largely answers the question “do standard trial designs in mild-to-moderate dementia produce clinically meaningful change?” It does not fully answer the newer biological question: “is there a coherent signal in biomarker-defined AD-spectrum populations at specific stages with stage-appropriate endpoints and mechanistically linked biomarkers?” That question motivates renewed attention to biomarker-enriched designs rather than repetition of mixed-syndrome paradigms. At the same time, signal dilution is unlikely to be the only explanation for historical inconsistency. Placebo-related influences, genuinely small treatment effects, and the possibility of small-study effects may also have contributed to the mixed pattern of the earlier literature.

## 7. Safety and Tolerability

Safety is frequently cited as a reason for the popularity of Ginkgo biloba preparations, but safety should be described more specifically than simply stating that tolerability is “similar to placebo.” Trial-based syntheses generally indicate no significant excess of overall adverse events or serious adverse events with standardized EGb 761 relative to placebo in monitored RCT settings [10,11,24,26]. Tolerability also appears broadly comparable at the level of treatment discontinuation, and some analyses have reported lower rates of adverse-event-related withdrawal with Ginkgo biloba than with placebo [11]. In addition, pooled analyses have not identified a consistent pattern of increased common adverse events; rather, selected events such as dizziness, tinnitus, and headache were, in some analyses, reported less frequently with EGb 761 than with placebo [10]. However, these findings should be interpreted within the confines of relatively short, controlled trials using standardized preparations. In real-world older adults, clinical risk assessment must still consider frailty, comorbidity, and polypharmacy, particularly concomitant antiplatelet or anticoagulant therapy, as well as the limited generalizability of trial-based safety findings to non-standardized supplement products [26]. Accordingly, the available literature supports good trial-based tolerability of standardized Ginkgo preparations, while not removing the need for individualized clinical caution in routine practice.

## 8. The Biomarker Pivot: Why Mixed Populations May Have Hidden a “Bit” of Signal

The most important development in AD research over the past decade is the shift toward biological definition and biomarker-based staging. Under contemporary biomarker frameworks, a major limitation of the historical literature becomes more apparent: clinically defined dementia cohorts likely included biologically heterogeneous populations [16,34,35]. This is particularly relevant for interventions expected to have small-to-modest effects, as such heterogeneity may have reduced the ability of earlier trials to detect a treatment signal. Therefore, biomarker-confirmed cohorts—particularly amyloid PET-positive MCI—provide a rational, albeit still preliminary, stress test for whether long-standing mechanistic plausibility can connect to coherent clinical and biomarker change, given that the currently available studies remain limited by small sample sizes, retrospective or secondary analytic designs, and limited independent replication. The pivot is not merely philosophical; it is methodological. If the historical program was optimized to produce ambiguity (mixed syndromes, variable endpoints, limited biological anchoring), biomarker-enriched designs are optimized to answer a sharper question: is there a reproducible signal when cohort biology, stage, and outcomes are aligned?

## 9. Biomarker-Confirmed Evidence in Amyloid PET-Positive MCI/AD

A key recent development is the report of Ginkgo monotherapy in amyloid PET-positive MCI incorporating both clinical outcomes and a plasma measure related to amyloid oligomerization tendency, the Multimer Detection System–Oligomerized Aβ (MDS-OAβ) assay [20,21]. The qualitative difference from classic trials lies in three features: pathology enrichment (amyloid positivity), earlier symptomatic stage (MCI), and inclusion of a mechanism-adjacent biomarker readout. Notably, the biomarker behaved directionally in a way that is difficult to attribute to nonspecific symptomatic fluctuation: MDS-OAβ decreased in the Ginkgo-treated group but increased in the comparison group not receiving Ginkgo, suggesting a divergence in the trajectory of oligomerization propensity rather than a mere cross-sectional difference. Interpreted conservatively, this pattern does not prove disease modification, but it does provide a biologically anchored context for modest clinical stability. In addition, these findings do not permit causal inference, and MDS-OAβ should currently be regarded as a mechanism-adjacent and still incompletely validated biomarker rather than a definitive surrogate of treatment response. This is consistent with the idea that, in an amyloid-confirmed prodromal population, treatment-associated clinical signals may coincide with a coherent shift in an amyloid-related process that is more proximal to synaptic toxicity than plaque burden alone. Add-on strategies in biomarker-confirmed AD attempt to answer a different but clinically relevant question: can Ginkgo meaningfully improve outcomes on top of standard symptomatic therapy in biologically confirmed disease? In amyloid PET-positive AD, adjunct-to-donepezil designs have reported aligned clinical outcomes alongside changes in the same oligomerization-related plasma biomarker, again implying that small clinical effects—when present—may be interpretable through biomarker movement rather than being inferred solely from symptom scales [22]. Importantly, this line of investigation extends beyond short-term biomarker change to longitudinal concordance between plasma oligomerization tendency and brain amyloid dynamics. In an 18-month retrospective amyloid PET-positive MCI cohort with serial MDS-OAβ measurements and paired amyloid PET, MDS-OAβ decreased over follow-up in the Ginkgo group but increased in the non-Ginkgo group, while global amyloid PET standardized uptake value ratio (SUVR) remained largely stable with Ginkgo and increased without Ginkgo [21]. Although this finding still requires independent replication, this type of triangulation—clinical trajectory plus a mechanism-adjacent blood biomarker plus longitudinal amyloid imaging—illustrates the central interpretive advance of biomarker-confirmed designs: they enable more insight into why outcomes diverge and in whom a modest clinical signal may be biologically coherent, rather than leaving efficacy debates to symptom scales alone [16,20,21,22]. Taken together, these observations suggest that restricting evaluation to biologically confirmed AD-spectrum cohorts may increase the likelihood of detecting a meaningful and interpretable treatment signal, if one exists. However, the current evidence remains preliminary and should be interpreted cautiously, given the relatively small sample sizes, the predominantly retrospective or secondary analytic nature of the available studies, the limited capacity for causal inference, the still incomplete validation of MDS-OAβ as a biomarker, and the fact that several of the cited biomarker-linked studies originate from the same research group, which further limits independent validation and generalizability; larger prospective studies from independent groups will be needed to confirm these findings.

## 10. Why “Stop Mixing Populations” Is a Testable Hypothesis

The long-standing mixed-population problem can be reframed as testable predictions. If benefits are contingent on amyloid-positive AD-spectrum biology, effect sizes should be more consistent in amyloid-positive MCI/AD than in mixed-syndrome cohorts; conversely, if effects are nonspecific or purely symptomatic across etiologies, biological enrichment should not systematically increase consistency. Similarly, if biomarker change is mechanistically relevant, biomarker movement should align directionally with clinical change more often in biologically defined cohorts. Testing these predictions requires adequate power, pre-specified subgroup plans, stage-appropriate endpoints, and transparent exposure characterization [5,16,23].

## 11. Practical Implications for Next-Generation AD-Focused Trials

**Figure 3:**
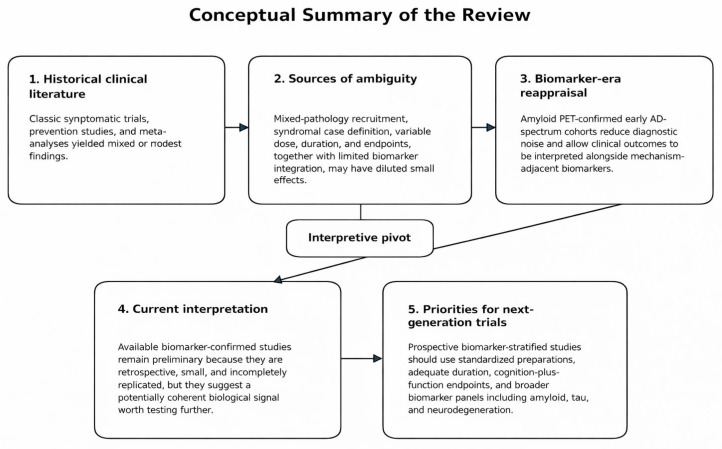
Conceptual summary of the review: from historical ambiguity to biomarker-informed cautious optimism in Ginkgo biloba research for Alzheimer’s disease. Historical trials and meta-analyses of Ginkgo biloba in dementia yielded mixed or modest findings, likely due in part to signal dilution from mixed populations, methodological heterogeneity, and limited biomarker integration. In the biomarker era, amyloid PET-confirmed early AD-spectrum cohorts offer a more interpretable setting for testing whether a modest clinical signal is biologically coherent. Although the current biomarker-confirmed evidence remains preliminary, it supports further evaluation in prospective biomarker-stratified trials.

A credible next-generation program in AD would align cohort definition (amyloid confirmation, and ideally tau/neurodegeneration staging), disease stage (early symptomatic MCI or very mild dementia), exposure certainty (standardized preparation with transparent characterization), and duration long enough to distinguish transient symptomatic fluctuation from sustained trajectory effects. Although the currently available biomarker-confirmed Ginkgo studies have primarily been conducted in amyloid PET-positive cohorts, future studies would ideally also incorporate tau-informed staging, given that tau pathology may be more closely linked to cognitive impairment and clinical progression. Endpoints should integrate cognition and function and include caregiver-relevant domains, while biomarker panels should include mechanism-adjacent readouts and broader neurodegeneration markers selected in relation to the hypothesized biological actions of Ginkgo biloba, including pathways related to oxidative stress, mitochondrial function, neuroinflammation, microcirculation, and amyloid-related processes. This approach may help clarify whether modest clinical signals are accompanied by biologically coherent changes across relevant pathways. Future biomarker-informed trials may also benefit from incorporating genotype stratification, as genetic background could contribute to treatment heterogeneity and help identify more responsive subgroups. The overarching point is that if the expected effect is modest, trial design must be optimized to detect modest effects—otherwise the literature will continue to generate internally consistent ambiguity (Figure 3).

## 12. Discussion

A fair reading of the classic clinical literature supports caution. Symptomatic trials show heterogeneous and often modest improvements, and prevention trials in broad older populations were convincingly null for reducing incident dementia/AD. Systematic reviews and meta-analyses repeatedly converge on “possible benefit versus placebo” alongside uncertainty driven by heterogeneity and debates about clinical meaningfulness. The recent critique concluding “not much” is therefore understandable for the historical paradigm [5]. Yet a biomarker-era, AD-specific lens reframes not only what we ask but what we can interpret: much of the earlier program was scale-driven, offering limited insight into why a subset of patients might stabilize or improve—an interpretive gap that is particularly salient for multi-component botanical interventions. Biomarker-enriched MCI/AD studies now test a sharper hypothesis under biologically anchored case definitions: whether modest clinical stability is accompanied by coherent movement in mechanism-adjacent biomarkers. In amyloid PET-positive MCI/AD, recent reports suggest that clinical preservation can coincide with directional changes in plasma oligomerization tendency, with MDS-OAβ decreasing in treated patients while increasing in comparison groups—an observation that, while not proof of disease modification, strengthens biological interpretability beyond symptom scales alone. If such patterns prove reproducible in larger biomarker-stratified studies, the field’s conclusion may shift from “not much” to “a bit.”—not as an argument for indiscriminate use, but as a rationale to stop mixing populations, standardize exposure, and design stage-appropriate, biomarker-anchored trials in early AD-spectrum disease.

## 13. Conclusions

Overall, the clinical evidence for Ginkgo biloba preparations in AD-spectrum disease remains heterogeneous. Nevertheless, recent biomarker-confirmed studies in amyloid PET-positive populations offer a more biologically interpretable framework and raise the possibility that a modest but coherent signal may have been obscured in earlier mixed-population studies. Although these findings remain limited by small sample sizes, retrospective or secondary analytic designs, and limited independent replication, they provide a reasonable basis for cautious optimism. Larger prospective biomarker-stratified trials with standardized preparations and clinically meaningful endpoints are needed to determine whether this emerging signal is reproducible and clinically meaningful.

## Figures and Tables

**Table 1:** Evidence map of Ginkgo biloba studies across the Alzheimer’s disease spectrum.

Evidence Cluster	Population	Biomarker Confirmation	Intervention (Typical)	Endpoints (Typical)	Main Finding (Overall)	Key Limitation/Interpretation Issue
Classic symptomatic RCTs (mixed dementia era)	AD + VaD/mixed dementia	None (syndromal diagnosis)	Standardized extract ~120–240 mg/day, ~22–26 weeks	Cognition, global impression, ADL, sometimes NPI	Mixed/modest effects	Population mixing; short duration; endpoint mismatch
Symptomatic RCTs in clinically diagnosed AD (AD-only trials)	Probable AD	None	Standardized extract (dose varies) vs. placebo	Cognition, global, ADL	Small/inconsistent	Clinical AD includes amyloid cases; sensitivity concerns
NPS-enriched dementia trials	Mild–moderate dementia with neuropsychiatric symptoms	None	EGb 761 240 mg/day, months	Cognition + NPI + ADL	More consistently positive (especially NPS/ADL)	Limited generalizability; non-biomarker-defined
Head-to-head/combination trials	AD dementia (clinical) ± NPS	None	Ginkgo vs donepezil or combination	Cognition, global, NPI, ADL	Suggestive/mixed	Limited power; background therapy confounding
Prevention trials (community older adults)	Older adults (broad population)	None	EGb 761, long follow-up	Incident dementia/AD; cognitive decline	Null for prevention	Not targeted to AD biology; dilution of signal
Systematic reviews/meta-analyses	Mixed dementia + AD	Usually none	Aggregated trials	Cognition, ADL, NPI, safety	Possible benefit but heterogeneous	Heterogeneity; preparation definition; clinical meaningfulness debate
Biomarker-confirmed MCI (amyloid PET+)	Amyloid PET+ MCI (prodromal AD)	Amyloid PET confirmed	Ginkgo 240 mg/day, 12 months	Cognition (K-MMSE), function (K-IADL), plasma MDS-OAβ	Positive signal (clinical stability + biomarker decrease)	Retrospective; needs prospective replication
Biomarker-confirmed AD (add-on)	Amyloid PET+ AD dementia	Amyloid PET confirmed	Ginkgo + donepezil	Clinical outcomes + MDS-OAβ	Suggestive positive alignment	Retrospective; sample size limitations

Evidence map of the principal study groups considered in this review across the Alzheimer’s disease spectrum, summarizing population, biomarker confirmation, intervention, endpoints, overall direction of findings, and major limitations. Abbreviations: AD, Alzheimer’s disease; VaD, vascular dementia; MCI, mild cognitive impairment; PET, positron emission tomography; K-MMSE, Korean Mini-Mental State Examination; ADL, activities of daily living; NPI, Neuropsychiatric Inventory; MDS-OAβ, Multimer Detection System–Oligomerized Aβ.

**Table 2:** Major bioactive component classes of Ginkgo biloba preparations and their proposed relevance to AD-related biological pathways.

Component Class	Representative Constituents	Proposed AD-Relevant Biological Actions	Interpretive Note
Flavonoid glycosides	Quercetin-, kaempferol-, and isorhamnetin-derived glycosides	Antioxidant and free-radical-scavenging effects; possible contribution to cellular stress resistance. Mechanistic links to mitochondrial, vascular, and synaptic function are biologically plausible but are more often described for the standardized extract as a whole rather than for isolated glycosides.	These effects are biologically plausible but do not by themselves establish clinical efficacy in AD.
Terpene lactones	Ginkgolides (A, B, C, J) and bilobalide	Possible modulation of neuroinflammation-related pathways and neuronal membrane/mitochondrial function; may contribute to vasoactive or microcirculatory effects at the extract level.	Likely relevant to the multimodal pharmacology of standardized extracts, but human AD-specific mechanistic evidence remains limited.
Flavonol aglycone-related metabolites	Downstream metabolites formed after flavonoid biotransformation	Potential contribution to systemic redox-related effects after metabolism, although relevance in human AD remains uncertain and may depend on bioavailability and metabolism.	This is a cautious, inferential mechanism rather than a directly established explanation in human AD.
Proanthocyanidin/polyphenolic fractions	Additional polyphenolic fractions, including proanthocyanidins, present in some standardized preparations	Possible antioxidant and vascular-supporting effects.	These fractions are less consistently emphasized in the clinical literature than flavonoid glycosides and terpene lactones, and their relative representation may vary across preparations.
Multi-component extract as administered in clinical studies	Pharmaceutical-grade standardized Ginkgo biloba extract as a composite exposure	Combined effects potentially involving oxidative stress modulation, mitochondrial support, microcirculatory regulation, synaptic resilience, and broader network-level biological effects.	The clinical literature evaluates the extract as a whole rather than isolated constituents; therefore, constituent-level mechanisms should be viewed as complementary rather than definitive explanations.

This table is intended to provide a mechanism-oriented interpretive framework rather than to imply that any single constituent has established disease-specific efficacy in Alzheimer’s disease.

**Table 3:** Clinician-oriented summary: when is a Ginkgo signal more likely to be detected in AD-spectrum studies?

Condition/Context	Where the “Signal” Is Most Often Observed	Overall Pattern	Practical Implication
High neuropsychiatric symptom (NPS) burden (higher baseline NPI)	NPI/behavioral outcomes, global impression, sometimes ADL	More consistently positive signals than cognition-only outcomes	In NPS-enriched patients, prioritize behavioral and functional targets rather than small cognitive deltas
Higher dose (240 mg/day) of a standardized, pharmaceutical-grade extract	Cognition + NPI + ADL (broader domain effects)	Signals reported more consistently than with lower/variable dosing	Standardize exposure at 240 mg/day when feasible and clearly report preparation
Adequate duration (≥~22–26 weeks)	Symptomatic outcomes (cognition, NPI, global, ADL)	Better detectability than very short trials; durability still uncertain	Ensure at least ~6 months to evaluate symptomatic benefit
Earlier symptomatic stage (MCI/very mild dementia)	Stability/less worsening; function; biomarker directionality	Effects often appear as stabilization rather than large improvement	Frame goals as maintenance/slower decline and use stage-appropriate endpoints
Later stage (mild–moderate dementia)	Mixed across domains	Smaller, heterogeneous effects; clinical meaningfulness debated	Set conservative expectations; endpoint selection becomes critical
Biomarker-confirmed AD-spectrum (e.g., amyloid PET+)	Clinical outcomes aligned with mechanism-adjacent biomarkers (e.g., MDS-OAβ)	Greater interpretability; evidence base still limited	Enrich cohorts biologically and pair clinical outcomes with biomarkers to reduce ambiguity
Population mixing (AD + VaD/mixed dementia; amyloid cases)	Any domain	Signal dilution → near-zero or inconsistent results	Avoid mixed-pathology recruitment when testing AD-specific hypotheses
Endpoint framework (cognition-only vs. cognition + function/caregiver-relevant)	Cognition + function/behavior/global	Integrated endpoints often provide more persuasive signals	Use cognition–function composites and caregiver-relevant outcomes, not cognition alone

This table summarizes clinical and design contexts in which Ginkgo biloba-associated signals have been more (or less) detectable across the AD spectrum, emphasizing factors that may amplify or dilute modest effects (population definition, dose, duration, stage, and endpoint selection). Abbreviations: AD, Alzheimer’s disease; ADL, activities of daily living; MCI, mild cognitive impairment; MDS-OAβ, Multimer Detection System–Oligomerized Aβ; NPI, Neuropsychiatric Inventory; NPS, neuropsychiatric symptoms; PET, positron emission tomography; VaD, vascular dementia.

## Data Availability

No new data were created or analyzed in this study.

## References

[1] Livingston G., Huntley J., Sommerlad A., Orgeta V., Costafreda S.G., Ames D., Ballard C., Banerjee S., Burns A., Cohen-Mansfield J. (2020). Dementia prevention, intervention, and care: 2020 report of the Lancet Commission. Lancet.

[2] Fox N.C., Belder C., Ballard C., Kales H.C., Mummery C., Caramelli P., Ebmeier K.P., Isaacson R.S., Karlawish J., Lovestone S. (2025). Treatment for Alzheimer’s disease. Lancet.

[3] Le Bars P.L., Katz M.M., Berman N., Itil T.M., Freedman A.M., Schatzberg A.F. (1997). A placebo-controlled, double-blind, randomized trial of an extract of Ginkgo biloba for dementia. JAMA.

[4] Schneider L.S., DeKosky S.T., Farlow M.R., Tariot P.N., Hoerr R., Kieser M. (2005). A randomized, double-blind, placebo-controlled trial of two doses of Ginkgo biloba extract in dementia of the Alzheimer’s type. Curr. Alzheimer Res..

[5] Necula B.R., Necula R.D., Petric P.S., Ifteni P.I., Irimie M., Dima L. (2024). EGb761 trials for mild-to-moderate dementia—What have we learned in the past 18 years?. Am. J. Ther..

[6] DeKosky S.T., Williamson J.D., Fitzpatrick A.L., Kronmal R.A., Ives D.G., Saxton J.A., Lopez O.L., Burke G., Carlson M.C., Fried L.P. (2008). Ginkgo biloba for prevention of dementia: A randomized controlled trial. JAMA.

[7] Snitz B.E., O’Meara E.S., Carlson M.C., Arnold A.M., Ives D.G., Rapp S.R., Saxton J., Lopez O.L., Dunn L.O., Sink K.M. (2009). Ginkgo biloba for preventing cognitive decline in older adults: A randomized trial. JAMA.

[8] Napryeyenko O., Borzenko I. (2007). Ginkgo biloba special extract in dementia with neuropsychiatric features: A randomised, placebo-controlled, double-blind clinical trial. Arzneimittelforschung.

[9] Herrschaft H., Nacu A., Likhachev S., Sholomov I., Hoerr R., Schlaefke S. (2012). Ginkgo biloba extract EGb 761^®^ in dementia with neuropsychiatric features: A randomised, placebo-controlled trial to confirm the efficacy and safety of a daily dose of 240 mg. J. Psychiatr. Res..

[10] Tan M.S., Yu J.T., Tan C.C., Wang H.F., Meng X.F., Wang C., Jiang T., Zhu X.C., Tan L. (2015). Efficacy and adverse effects of Ginkgo biloba for cognitive impairment and dementia: A systematic review and meta-analysis. J. Alzheimer’s Dis..

[11] Hashiguchi M., Ohta Y., Shimizu M., Maruyama J. (2015). Meta-analysis of the efficacy and safety of Ginkgo biloba extract for the treatment of dementia. J. Pharm. Health Care Sci..

[12] Riepe M., Hoerr R., Schlaefke S. (2025). Ginkgo biloba extract EGb 761 is safe and effective in the treatment of mild dementia: A meta-analysis of patient subgroups in randomised controlled trials. World J. Biol. Psychiatry.

[13] Pfuhlmann K., Koch A.K., Langhorst J. (2025). Ginkgo biloba leaf extract EGb 761^®^ for the treatment of various diseases: Overview of systematic reviews. Phytomedicine.

[14] Bohlken J., Hajek A., Burkart M., Kostev K. (2025). Ginkgo biloba extract prescriptions are associated with slower progression of dementia severity: Analysis of longitudinal real-world data. Brain Sci..

[15] European Directorate for the Quality of Medicines & HealthCare (2012). Ginkgo dry extract, refined and quantified (Monograph 04/2008:1827). European Pharmacopoeia (Ph. Eur.).

[16] Jack C.R., Bennett D.A., Blennow K., Carrillo M.C., Dunn B., Haeberlein S.B., Holtzman D.M., Jagust W., Jessen F., Karlawish J. (2018). NIA-AA Research Framework: Toward a biological definition of Alzheimer’s disease. Alzheimer’s Dement..

[17] Kim S.H. (2019). A therapeutic strategy for Alzheimer’s disease focused on immune-inflammatory modulation. Dement. Neurocogn. Disord..

[18] Morató X., Marquié M., Tartari J.P., Lafuente A., Abdelnour C., Alegret M., Jofresa S., Buendía M., Pancho A., Aguilera N. (2023). A randomized, open-label clinical trial in mild cognitive impairment with EGb 761 examining blood markers of inflammation and oxidative stress. Sci. Rep..

[19] Lopez O.L., Chang Y., Ives D.G., Snitz B.E., Fitzpatrick A.L., Carlson M.C., DeKosky S.T., Saxton J., Rapp S.R., Arnold A.M. (2019). Blood amyloid levels and risk of dementia in the Ginkgo Evaluation of Memory Study (GEMS): A longitudinal analysis. Alzheimer’s Dement..

[20] Yang Y.S., Koo M.S., Kwak Y.T. (2025). Efficacy of Ginkgo biloba extract in amyloid PET-positive patients with mild cognitive impairment. Front. Neurol..

[21] Yang Y., Kwak Y.T. (2026). Ginkgo biloba and longitudinal changes in amyloid PET and plasma Aβ oligomerization in amyloid-positive MCI: A retrospective analysis. J. Alzheimer’s Dis..

[22] Yang Y.S., Koo M.S., Kwak Y.T. (2025). Efficacy of Ginkgo biloba as an adjunct to donepezil in amyloid PET-positive Alzheimer’s patients. Front. Neurol..

[23] Weinmann S., Roll S., Schwarzbach C., Vauth C., Willich S.N. (2010). Effects of Ginkgo biloba in dementia: Systematic review and meta-analysis. BMC Geriatr..

[24] Gauthier S., Schlaefke S. (2014). Efficacy and tolerability of Ginkgo biloba extract EGb 761^®^ in dementia: A systematic review and meta-analysis of randomized placebo-controlled trials. Clin. Interv. Aging.

[25] Wolsko P.M., Solondz D.K., Phillips R.S., Schachter S.C., Eisenberg D.M. (2005). Lack of herbal supplement characterization in published randomized controlled trials. Am. J. Med..

[26] McKeage K., Lyseng-Williamson K.A. (2018). Ginkgo biloba extract EGb 761^®^ in the symptomatic treatment of mild-to-moderate dementia: A profile of its use. Drugs Ther. Perspect..

[27] Tabassum N.E., Das R., Lami M.S., Islam M.A., Karim M.R., Rahman M.A., Uddin M.J., Hossain M.S., Hasan M.R., Hosen M.J. (2022). Ginkgo biloba: A treasure of functional phytochemicals with multimedicinal applications. Evid. Based Complement. Alternat. Med..

[28] Janssen I.M., Sturtz S., Skipka G., Zentner A., Velasco Garrido M., Busse R. (2010). Ginkgo biloba in Alzheimer’s disease: A systematic review. Wien. Med. Wochenschr..

[29] Ihl R., Tribanek M., Bachinskaya N. (2011). Efficacy and safety of a once-daily formulation of Ginkgo biloba extract EGb 761^®^ in dementia with neuropsychiatric features: A randomized controlled trial. Int. J. Geriatr. Psychiatry.

[30] Mazza M., Capuano A., Bria P., Mazza S. (2006). Ginkgo biloba and donepezil: A comparison in the treatment of Alzheimer’s dementia in a randomized placebo-controlled double-blind study. Eur. J. Neurol..

[31] Yancheva S., Ihl R., Nikolova G., Panayotov P., Schlaefke S., Hoerr R. (2009). Ginkgo biloba extract EGb 761(R), donepezil or both combined in the treatment of Alzheimer’s disease with neuropsychiatric features: A randomised, double-blind, exploratory trial. Aging Ment. Health.

[32] Birks J., Grimley Evans J. (2009). Ginkgo biloba for cognitive impairment and dementia. Cochrane Database Syst. Rev..

[33] Wieland L.S., Ludeman E., Chi Y., Feinberg T.M., Chen I.H., Chen K.H., Wang C.C., Thiyagarajan J.A., Nance M., Hempel S. (2026). Ginkgo biloba for cognitive impairment and dementia. Cochrane Database Syst. Rev..

[34] Ossenkoppele R., Jansen W.J., Rabinovici G.D., Knol D.L., van der Flier W.M., van Berckel B.N.M., Visser P.J., Amyloid PET Study Group (2015). Prevalence of amyloid PET positivity in dementia syndromes: A meta-analysis. JAMA.

[35] Jansen W.J., Janssen O., Tijms B.M., Vos S.J.B., Ossenkoppele R., Visser P.J., Hansson O., Frisoni G.B., Villemagne V.L., Teunissen C.E. (2022). Prevalence estimates of amyloid abnormality across the Alzheimer disease clinical spectrum. JAMA Neurol..

